# Electric Characteristic Enhancement of an AZO/Si Schottky Barrier Diode with Hydrogen Plasma Surface Treatment and Al_x_O_x_ Guard Ring Structure

**DOI:** 10.3390/ma11010090

**Published:** 2018-01-08

**Authors:** Chien-Yu Li, Min-Yu Cheng, Mau-Phon Houng, Cheng-Fu Yang, Jing Liu

**Affiliations:** 1 Institute of Microelectronics, Department of Electrical Engineering, National Cheng Kung University, No. 1, University Road, Tainan 701, Taiwan; q18021014@mail.ncku.edu.tw (C.-Y.L.); q16044082@mail.ncku.edu.tw (M.-Y.C.); 2 Department of Chemical and Materials Engineering, National University of Kaohsiung, No. 700, Kaohsiung University Road, Nan-Tzu District, Kaohsiung 811, Taiwan; cfyang@nuk.edu.tw; 3 School of Information Engineering, Jimei University, Xiamen 361021, China

**Keywords:** Schottky barrier diodes, hydrogen plasma, guard ring, atomic layer deposition, rapid thermal annealing

## Abstract

In this study, the design and fabrication of AZO/n-Si Schottky barrier diodes (SBDs) with hydrogen plasma treatment on silicon surface and Al_x_O_x_ guard ring were presented. The Si surface exhibited less interface defects after the cleaning process following with 30 w of H_2_ plasma treatment that improved the switching properties of the following formed SBDs. The rapid thermal annealing experiment also held at 400 °C to enhance the breakdown voltage of SBDs. The edge effect of the SBDs was also suppressed with the Al_x_O_x_ guard ring structure deposited by the atomic layer deposition (ALD) at the side of the SBDs. Experimental results show that the reverse leakage current was reduced and the breakdown voltage increased with an addition of the Al_x_O_x_ guard ring. The diode and fabrication technology developed in the study were applicable to the realization of SBDs with a high breakdown voltage (>200 V), a low reverse leakage current density (≤72 μA/mm^2^@100 V), and a Schottky barrier height of 1.074 eV.

## 1. Introduction

A Schottky barrier diode (SBD) can be simply formed by a semiconductor substrate coming in contact with a metal electrode. It is a majority-carrier device that is mainly used in high-frequency applications, and it provides the advantage of fast reverse recovery without the minority-carrier stored charge that is observed in p–n rectifiers. In most of these devices, using a power rectifier with high-speed switching capability is indispensable. However, due to their low breakdown voltage characteristic, SBDs have rarely been applied to high-power devices. Several studies have investigated the causes of the low breakdown voltage issue. Most of these studies have indicated that the edge effect and the interface trap cause the low breakdown voltage and leakage current [1,2,3,4]. In SBDs, voltage drop under forward bias (V_F_) and leakage current density under reverse bias (J_R_) are the main concerns related to power applications, which strongly depend on the Schottky barrier height (SBH). Therefore, developing an SBD with a high breakdown voltage (V_BD_), low reverse leakage current density (J_R_), and low forward voltage drop (V_F_) is essential in the electronic market. Generally, a large SBH leads to a low J_R_ but a high V_F_. Moreover, V_BD_ is strongly influenced by the distribution of the surface electric field of the devices under reverse bias. There have been several studies discussed the edge effect that caused the leakage current [5]. The guard ring structure of p^+^ poly Si and metal oxide were studied to suppress the leakage current of the SBDs [6,7,8]. The interface between the substrate with existed multiple defects can also be removed by a treating plasma cleaning process and an annealing process [9,10,11]. Low-temperature processes are also important for the semiconductor industry because they help avoid unintentional diffusion effects and unwanted chemical reactions. An AZO/n-Si SBD fabricated using a simple process and low-cost material has the potential to succeed in the electronic commerce market [12,13]. In this paper, the design and fabrication of AZO/n-Si SBDs through the H_2_ plasma treatment of the Si surface and the introduction of an Al_x_O_x_ guard ring are presented.

## 2. Fabrication of AZO/Si Diode

To fabricate the AZO/Si SBDs, a single-crystalline Si wafer of (100) direction was first phosphorus doped with a resistivity of 1–10 cm. The thickness of the Si wafer was 525 μm. The resultant Si substrates were then wet-process treated. Specifically, the Si substrates were dipped in a buffered oxide etch (BOE) solution for 1 min to remove the native oxide. Thereafter, they were dipped in a sulfuric and hydrogen peroxide mixture (SPM) solution that consisted of 80% H_2_SO_4_ and 20% H_2_O_2_ for 5 min at 120 °C. The SPM process was performed to remove organic contamination on the surface of the Si substrates. Subsequently, the treated substrates were dipped in an ammonia and hydrogen peroxide mixture (APM) solution that consisted of 4% of NH_4_OH and 4.2% of H_2_O_2_ for 5 min at 70 °C in a constant temperature environment. The APM process has been demonstrated to effectively remove particles. The Si substrates were then dipped in a hydrochloric acid and peroxide mixture (HPM) solution that consisted of 4.5% of HCl and 3.75% of H_2_O_2_ for 5 min at 60 °C. The HPM process was performed to remove metal impurities. Finally, the Si substrates were again dipped in the BOE solution for 1 min. At the end of each process, the substrates were rinsed with DI water and dried with N_2_ gas.

**Figure 1:**
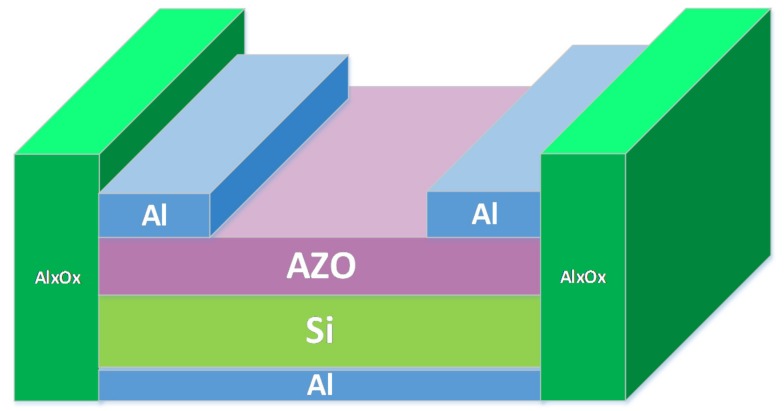
The structure of the AZO/Si diode with Al_x_O_x_ guard ring.

After all of the Si substrates were thoroughly cleaned using the wet etching process, they were treated with H_2_ plasma through microwave plasma chemical vapor deposition for 10, 30, and 60 s at room temperature. The H_2_ plasma power was 30 W at 1.4 torr. Subsequently, an n-type AZO thin film was deposited on the Si substrates to obtain a strong Schottky contact through radio frequency (RF) magnetron sputtering. The deposition of the AZO thin film was performed in an Ar (purity: 99.99%) atmosphere, with the deposition pressure maintained at 5 × 10^−3^ torr and the RF power controlled at 100 W. During deposition, the temperature of the substrates was maintained at 200 °C. After deposition, the Al layer served as the front and back electrodes to provide strong ohmic contact. Finally, an Al_x_O_x_ guard ring was added to the AZO/n-Si SBDs by using atomic layer deposition (ALD), followed by annealing in a N_2_ atmosphere at 400 °C for 30 min. A high-purity N_2_ (purity: 99.999%) atmosphere was utilized to carry the precursors and purge the by-products. The deposition pressure was kept constant at 1.4 torr. The structure of the AZO/Si diode was shown in the Figure 1. The size of the Si substrate was 1 × 1 cm^2^, with approximately 39 nm AZO deposited on the Si substrate. The Al electrodes were defined in 1 × 0.3 cm^2^ with the utilization of metal mask. The guard ring structure was deposited with metal mask in 1 × 0.2 cm^2^.

In this study, the contact angle of the Si substrates was determined through water contact angle measurement. Additionally, energy dispersive X-ray spectrometry and grazing-incidence X-ray diffraction (XRD, Rigaku, Tokyo, Japan) were performed to determine the crystalline properties of the AZO thin film by using Cu Kα radiation and a scanning angle (2θ) range of 20°–80°. Ultraviolet-visible spectroscopy was also conducted to determine the transmittance of the AZO thin film, and transmission electron microscopy (TEM, JEOL, Tokyo, Japan) was used to examine the grain sizes and structure of the poly-Si films. Conductive atomic force microscopy (Bruker, Billerica, MA, USA) was used to detect the surface height and current distribution, which could then be applied to detect the leakage current of the AZO/Si SBDs. Finally, Agilent B1500 (Agilent, Santa Clara, CA, USA) was used to measure the current density–voltage (J–V) characteristic of the fabricated SBDs.

## 3. Results and Discussion

In this section, we discuss the electrical properties of the fabricated AZO/Si SBDs. First, the deposition results of the AZO thin film, which affects the electrical properties of the diodes, are discussed. The switching characteristics of the diodes were observed using TEM images, C–V images, and J–V images; the H_2_ plasma treatment of the Si substrate surfaces was also observed using J−V images. Notably, a weak leakage current of the reverse bias and an exponential increment of the forward bias current were the characteristics of the rectifying contacts. Thermionic emission theory indicates that the current density in an SBD is given by the Equation (1) [14]:

(1) $$J=J_{s}\left(e^{-\frac{qV}{nkT}}-1\right)$$

**Figure 2:**
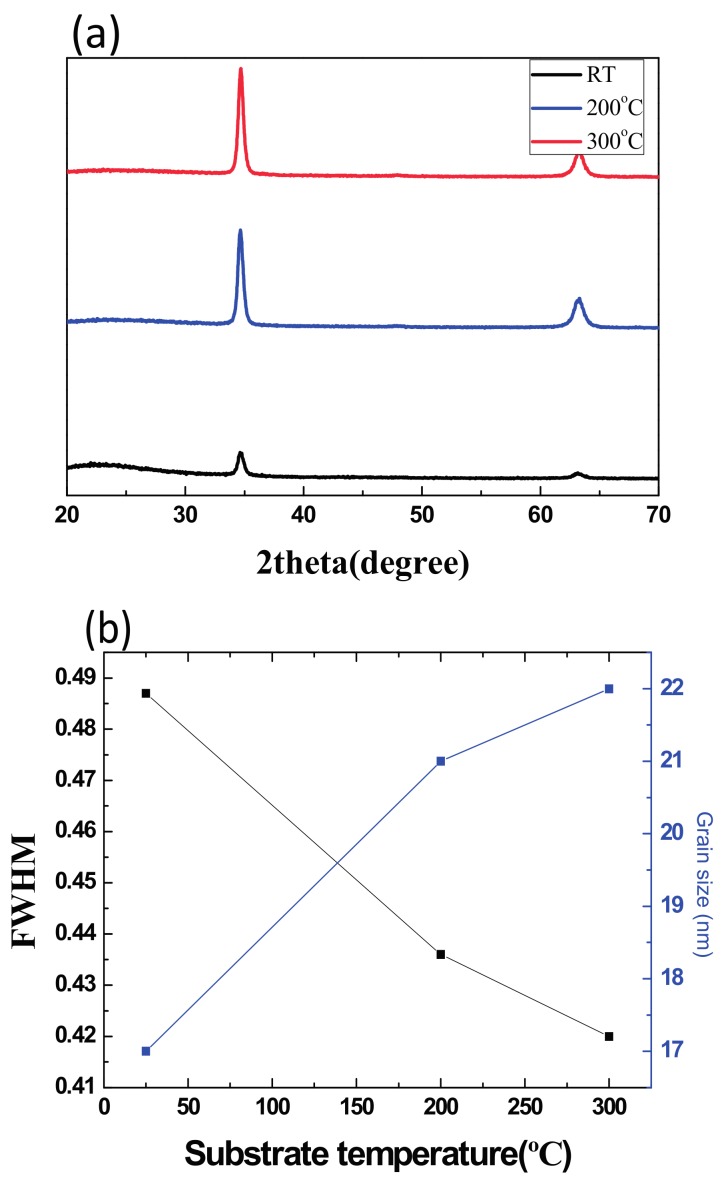
(**a**) The X-ray diffraction (XRD) measurement; (**b**) FHWM and grain size of AZO thin film deposited in room temperature, 200, and 300 °C.

Additionally, the saturation current density is given by

(2) $$J_{0}=A^{*}T^{2}e^{-\frac{q\phi_{B}}{kT}}$$

where A* is the effective Richardson constant, *T* is the temperature, *q* is the electronic charge, *k* is the Boltzman constant, *V* is the forward bias, *n* is the ideality factor, *J*_0_ is the saturation current density, and *φ_B_* is the effective barrier height. Notably, *J*_0_ is derived from the straight line intercept of *ln*(*J*) at zero bias, and *φ_B_* is calculated by Equation (2). The utility of the Al_x_O_y_ guard ring on the side of the SBDs was also examined. The cleanliness of the Si substrates and the crystallization of the AZO substantially affect the electrical properties of SBDs. Hence, in this study, we performed AZO deposition at three temperatures: room temperature, 200, and 300 °C. According to the X-ray diffraction measurement results in Figure 2a, a higher zinc oxide peak of (002) and a relatively lower peak of (103) were detected when AZO thin film deposition was performed at 200 °C. The full width at half maximum (FWHM) of the AZO (002) peak reduced with the raised of the deposition temperature, indicating the improved quality of crystallization. The calculated grain size of the AZO under 200 °C showed approximately size with 300 °C of 21 and 22 nm in the following figure.

**Figure 3:**
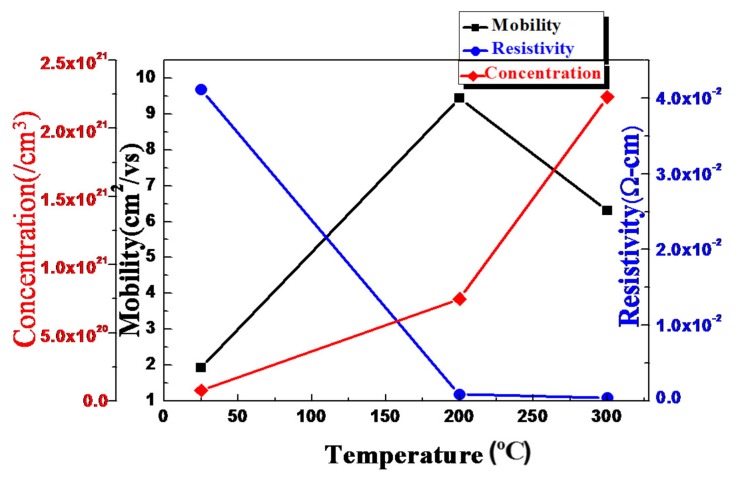
The Hall measurement of AZO thin film deposited in room temperature, 200, and 300 °C, showing the mobility, resistivity, and carrier concentration.

The improvement of the AZO crystallization quality also in concert with the Hall measurement result. The carrier concentration was up to 10^20^ order at 200 °C deposition which providing more conductivity. The resistivity, mobility, and carrier concentration of the AZO thin film, calculated using the Hall measurement, are presented in Figure 3. Notably, the carrier concentration of the AZO thin film deposited at 300 °C was higher than that of the film deposited at 200 °C. However, the higher ratio of Al to Z in the AZO thin film led to greater ionized impurity scattering and lower mobility, and the conductivity of the AZO thin film deposited at room temperature was too low for the film to be applied on the diode.

**Figure 4:**
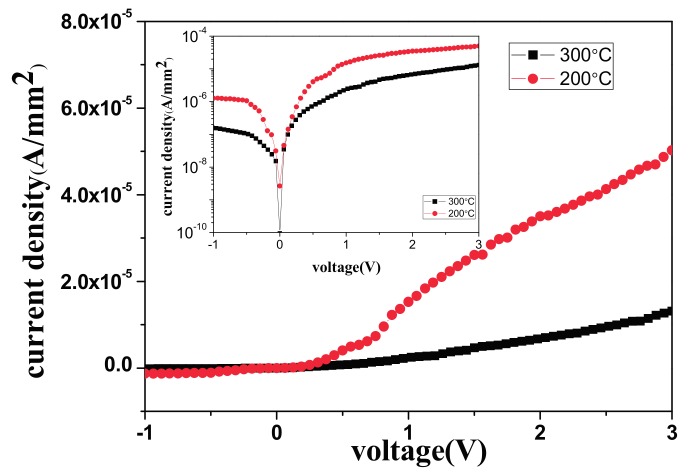
The J–V figure of 200 °C/300 °C AZO thin film deposited on Si substrate demonstrated a lower turn on voltage on 200 °C AZO/Si Schottky barrier diodes (SBDs).

Figure 4 reveals the experimental semi-log J–V characteristics of the AZO/n-Si SBDs at room temperature. The reverse saturation current density of the AZO/Si SBDs deposited at 300 °C was 1.18 × 10^−8^ A/mm^2^, which is lower than that of the AZO/Si SBDs deposited at 200 °C (5.78 × 10^−8^ A/mm^2^). However, the AZO/Si SBDs deposited at 200 °C had a lower turn on voltage than the AZO/Si SBDs deposited at 300 °C. 

**Figure 5:**
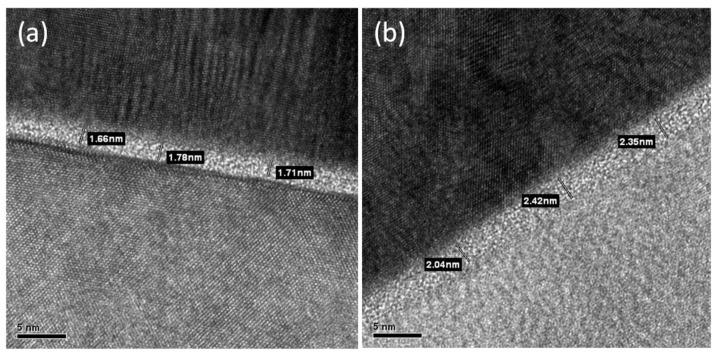
Transmission electron microscopy (TEM) figures of (**a**) 200 °C and (**b**) 300 °C AZO thin film deposited on Si substrate showed 1.7 and 2.3 nm SiO_x_ at the interface of AZO and Si substrate.

Differences between the AZO/Si SBDs deposited at 200 °C and those deposited at 300 °C are also evident in the TEM images shown in Figure 5. In particular, the SiO_x_ layer in the AZO/Si intermediate region was approximately 1.7 nm in the AZO/Si SBDs deposited at 200 °C (Figure 5a) and 2.3 nm in the AZO/Si SBDs deposited at 300 °C (Figure 5b). A thicker SiO_x_ layer decreased the reverse tunneling saturation current due to the addition of the effective barrier height, as shown in Figure 4.

A native oxide layer and metal particles on the surface of the Si substrates affect the SBD breakdown voltage. Although the Si substrates were thoroughly cleaned using the wet etching process, the ultrathin oxide layer formed during the subsequent process of AZO deposition on the substrate surface. The ultrathin oxide layer between Si and AZO causes tunneling of carrier and leakage current [15]. The interface trap between Si and SiO_2_, which was caused by the dangling bonds from the oxide layer, metal impurities, and the hot carrier, consumed the electronic charge and affected the I–V properties.

**Figure 6:**
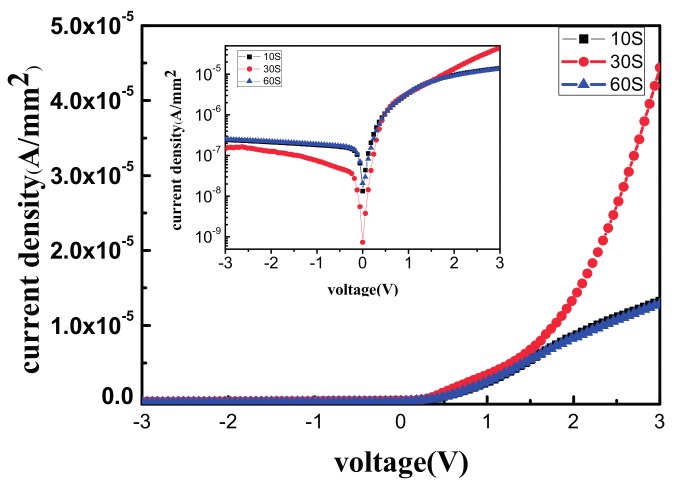
The J–V figure of 10 s/30 s/60 s H_2_ plasma treated on Si substrate demonstrated 4.43 × 10^−9^, 2.27 × 10^−9^, and 4.85 × 10^−9^ A/mm^2^ reverse saturation current density of the SBDs.

To overcome the aforementioned issues, we treated the Si substrate surfaces with H_2_ plasma to remove the native oxide layer and eliminate the dangling bonds [10,11]. The H_2_ plasma power was set to 30 W for 10, 30, and 60 s at room temperature. As depicted in Figure 6, this figure indicates that the saturation current density *J*_0_, which was analyzed by extrapolation to the forward bias of 0 V, was 4.43 × 10^−9^, 2.27 × 10^−9^, and 4.85 × 10^−9^ A/mm^2^, respectively. By substituting *J*_0_ in Equation (2), the SBH was determined to be 0.92, 0.93, and 0.85 eV, respectively. Our results demonstrated that the SBD with a Si substrate treated with H_2_ plasma for 30 s had the lowest reverse current density and the highest forward current.

**Figure 7:**
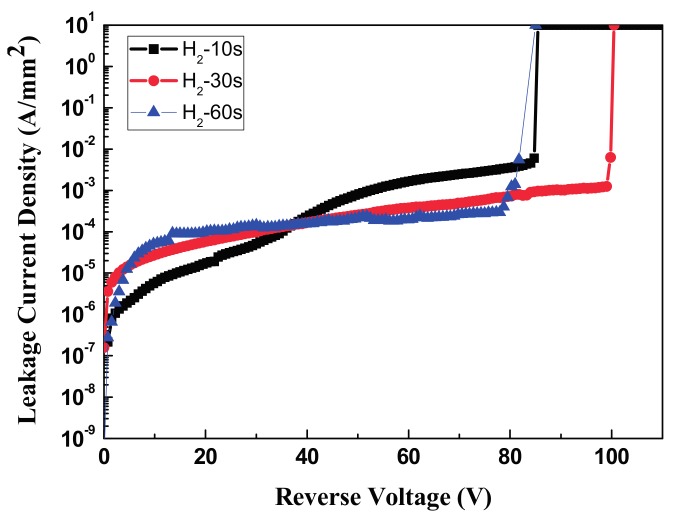
The J–V figure of 10 s/30 s/60 s H_2_ plasma treated on Si substrate presented the SBDs breakdown voltage of 85 v/100 v/81 v.

With an appropriate amount of power, the H_2_ plasma treatment effectively removed the oxide layer; however, if the H_2_ plasma treatment used too much power or too much time, the Si surface was damaged and defects were formed in the surface. After H_2_ plasma treated on the Si surface for 10, 30, 60 s, the breakdown voltages of the following formed AZO/n-Si SBDs increased to 85, 100, and 81 V, as revealed in Figure 7. 

**Figure 8:**
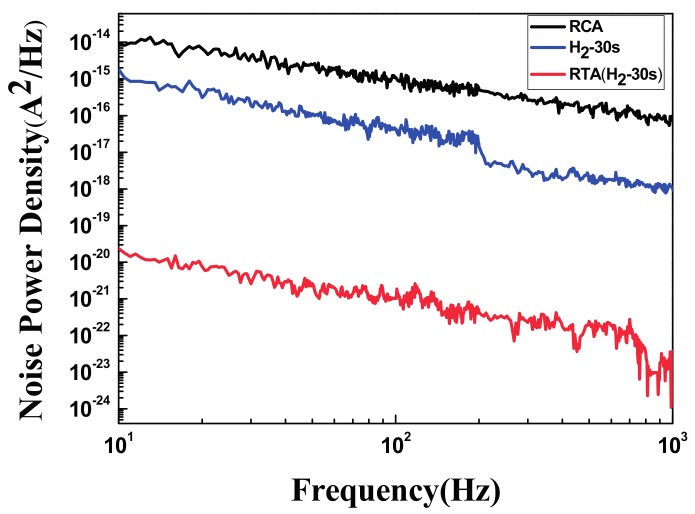
The flicker noise measurement of the H_2_ plasma treated sample demonstrated a lower noise power density than the sample that only treated with Radio Corporation of America (RCA) standard cleaning process. The Rapid Thermal Annealing (RTA) treated sample can further suppress the flicker noise and improved the interface of Si substrate.

Notably, the defects between the interface of the Si and the oxide layer trapped and detrapped the carriers and produced flicker noise. A subsequent annealing process would rearrange the atoms and decrease the number of defects on the Si surface. The flicker noise measurement was held under the constant current of 400 μA. The results of our flicker noise analysis are presented in Figure 8, and they show that the number of defects at the interface between the oxide layer and the Si surface considerably decreased following the H_2_ plasma treatment and annealing process. This result confirmed that the H_2_ plasma treatment and annealing decrease the number of defects at the interface.

**Figure 9:**
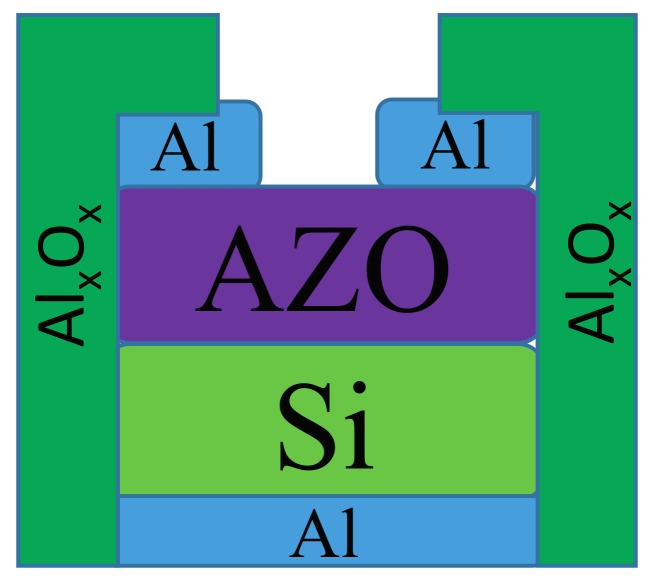
The structure of the AZO/Si SBD with Al_x_O_x_ guard ring.

The reverse current is the most critical characteristic influencing the switching behavior of SBDs. Notably, the leakage current at the edge of SBDs, which is caused by the deep edge of the metal electrode, comprises most of the reverse current. To eliminate the adverse effect of the leakage current, some researchers have included a guard ring when fabricating SBDs. In most previous studies, the guard rings used consisted of a deep doped P-type diffusion area. However, in the present study, Al_x_O_x_ was utilized as the guard ring and was deposited around the AZO/Si SBDs by using ALD. The structure of the AZO/Si SBDs with the guard ring is shown in Figure 9. The green vertical part of Figure 9 is the Al_x_O_x_ layer. The deposition was processed through the metal mask covering on the middle of the diode. The exposed part at the side of diode was then deposited on the Al_x_O_x_ layer.

**Figure 10:**
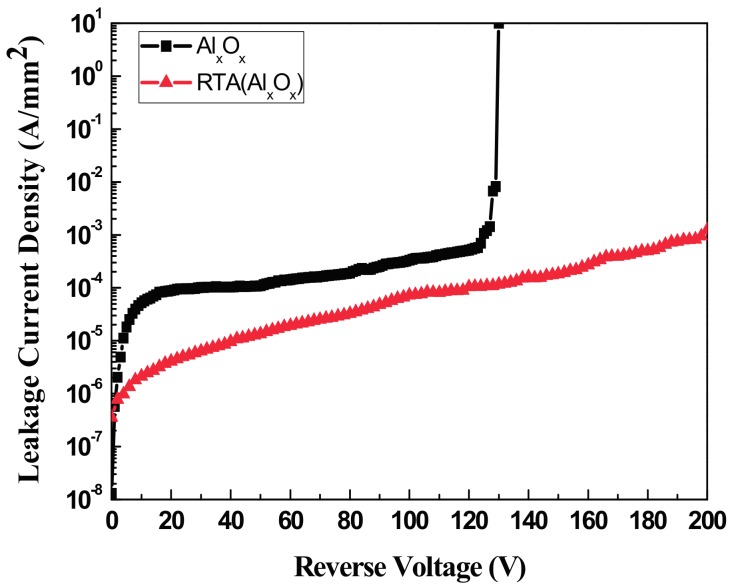
The breakdown voltage of the SBDs with guard ring structure raised to 130 V before the RTA annealing process and further increased to over 200 V after the RTA annealing.

The Al_x_O_x_ guard ring isolated the leakage current around the electrode and enabled the SBDs to have the switching properties of a high breakdown voltage and low reverse current. As illustrated in Figure 10, the breakdown voltage of the unannealed SBDs increased to 130 V when the Al_x_O_x_ guard ring was added; these SBDs also had a lower reverse current. However, following the rapid thermal annealing process, the breakdown voltage of these SBDs were further improved to over 200 V.

**Figure 11:**
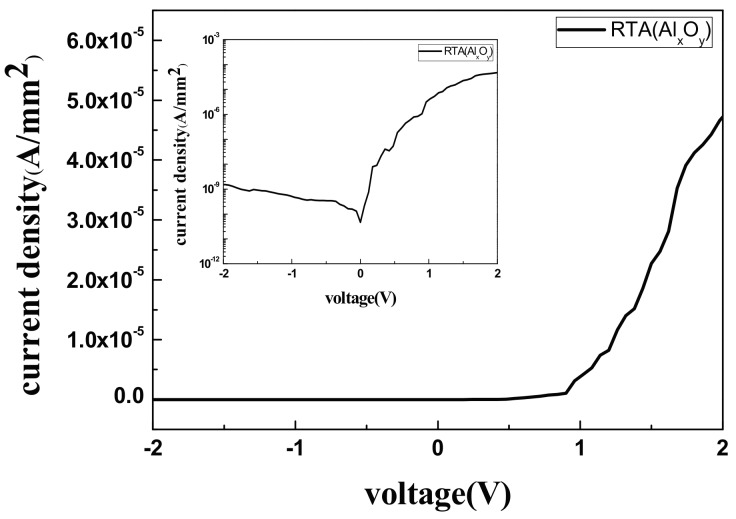
The J–V figure of the SBDs with Al_x_O_x_ guard ring showed a 4.62 × 10^−11^ A/mm^2^ saturation current density (*Js*).

The J–V results of the SBDs with Al_x_O_x_ guard ring presented in Figure 11 illustrate a reverse saturation current density (*Js*) of 4.62 × 10^−11^ A/mm^2^ and a barrier height of 1.074 eV. Therefore, adding a guard ring to SBDs reduces the reverse current and improves the forward bias.

## 4. Conclusions 

In this study, we successfully fabricated AZO/Si SBDs with a high breakdown voltage. By modifying the AZO deposition parameters, we were able to improve the crystallization of the AZO and the saturation current density (*Js*) of the SBDs. Additionally, we demonstrated that the H_2_ plasma treatment substantially reduced the number of defects at the interface between the oxide layer and the Si surface, as well as reduced the current noise power spectral density by 10 times. Prior to annealing, the AZO/Si SBDs had the following switching properties: a breakdown voltage of 100 V, leakage current density of 2.27 × 10^−9^ A/mm^2^, and SBH of 0.923 eV. The current noise power spectral density of the annealed AZO/Si SBDs was also 10^6^ times less than that of the unannealed SBDs. Finally, adding a guard ring and proceeding the Rapid Thermal Annealing (RTA) at 400°C was found to further increase the breakdown voltage to over 200 V and reduce the leakage current density to less than 72 μA/mm^2^ at the 100 V bias. The reverse saturation current density was also reduced to 4.62 × 10^−11^ A/mm^2^, and the SBH was increased to 1.074 eV.
